# Characteristics of U.S. Adults with Usual Daily Folic Acid Intake above the Tolerable Upper Intake Level: National Health and Nutrition Examination Survey, 2003–2010

**DOI:** 10.3390/nu8040195

**Published:** 2016-04-01

**Authors:** Angela M. Orozco, Lorraine F. Yeung, Jing Guo, Alicia Carriquiry, Robert J. Berry

**Affiliations:** 1 Division of Congenital and Developmental Disorders, National Center on Birth Defects and Developmental Disabilities, Centers for Disease Control and Prevention, Atlanta, GA 30341, USA; aorozco1@jhmi.edu (A.M.O.); shashagj@gmail.com (J.G.); rjb1@cdc.gov (R.J.B.); 2 David Geffen School of Medicine at University of California-Los Angeles, Los Angeles, CA 90095, USA; 3 Department of Statistics, Iowa State University, Ames, IA 50011, USA; alicia@iastate.edu

**Keywords:** NHANES, folic acid, dietary supplements

## Abstract

The Food and Drug Administration mandated that by 1998, all enriched cereal grain products (ECGP) be fortified with folic acid in order to prevent the occurrence of neural tube defects. The Institute of Medicine established the tolerable upper intake level (UL) for folic acid (1000 µg/day for adults) in 1998. We characterized U.S. adults with usual daily folic acid intake exceeding the UL. Using NHANES 2003–2010 data, we estimated the percentage of 18,321 non-pregnant adults with usual daily folic acid intake exceeding the UL, and among them, we calculated the weighted percentage by sex, age, race/ethnicity, sources of folic acid intake, supplement use and median usual daily folic acid intakes. Overall, 2.7% (standard error 0.6%) of participants had usual daily intake exceeding the UL for folic acid; 62.2% were women; 86.3% were non-Hispanic whites; and 98.5% took supplements containing folic acid. When stratified by sex and age groups among those with usual daily folic acid intake exceeding the UL, 20.8% were women aged 19–39 years. Those with usual daily intake exceeding the folic acid UL were more likely to be female, non-Hispanic white, supplement users or to have at least one chronic medical condition compared to those not exceeding the folic acid UL. Among those with usual daily folic acid intake exceeding the UL who also took supplements, 86.6% took on average >400 µg of folic acid/day from supplements. Everyone with usual daily folic acid intake exceeding the UL consumed folic acid from multiple sources. No one in our study population had usual daily folic acid intake exceeding the UL through consumption of mandatorily-fortified enriched cereal grain products alone. Voluntary consumption of supplements containing folic acid is the main factor associated with usual daily intake exceeding the folic acid UL.

## 1. Introduction

Folic acid reduces a woman’s risk of having a child affected by a neural tube defect, such as spina bifida [1,2]. In 1996, the Food and Drug Administration mandated that all enriched cereal grain products (ECGP) be fortified with folic acid by January 1998 [3]. The Institute of Medicine (IOM) conducted a comprehensive review of folic acid safety and concluded that progression of neuropathy among vitamin B-12-deficient individuals receiving folic acid doses ≥5000 µg/day was a potential adverse effect of folic acid. The IOM identified 5000 µg/day as the lowest observed adverse effect level (LOAEL) for folic acid and then established a tolerable upper intake level (UL) (1000 µg/day for adults) for folic acid by dividing the LOAEL by an uncertainty factor of five [4].

Studies have found that less than 3% of U.S. adults had usual daily folic acid intake exceeding the UL post-fortification [5,6]; among those aged >50 years, approximately 5% had usual daily folic acid intake exceeding the UL [7]. To date, no studies have described the characteristics of adults with usual daily intake of folic acid exceeding the UL. We aim to characterize U.S. adults with usual daily intake of folic acid exceeding the UL and describe their sources of folic acid consumption.

## 2. Methods

### 2.1. Study Population

We obtained data from the National Health and Nutrition Examination Survey (NHANES), a stratified multistage probability survey designed to represent the civilian, noninstitutionalized U.S. population. The National Center for Health Statistics (NCHS) collected data via household interviews and physical examinations. Detailed information regarding NHANES is available elsewhere [8,9,10,11]. The survey was reviewed and approved by the NCHS research ethics review board, and participants provided written informed consent prior to participation. We selected non-pregnant adults aged ≥19 years from NHANES 2003–2010.

**Figure 1:**
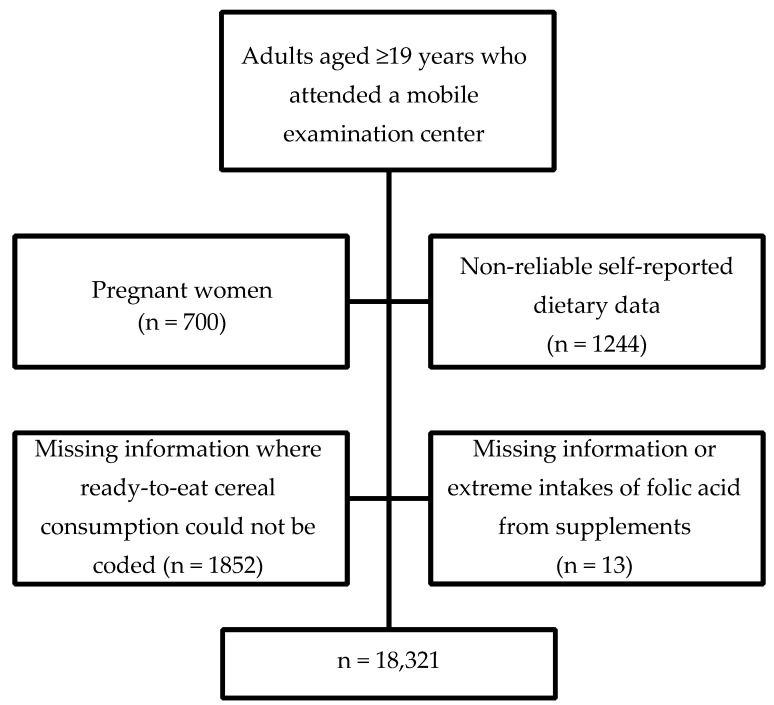
Exclusion criteria to select study population from which to assess for non-pregnant U.S. adults with usual daily folic acid intake exceeding the tolerable upper level for folic acid: National Health and Nutrition Examination Survey (NHANES), 2003–2010.

The overall unweighted examination response rates for adults in NHANES 2003–2004, 2005–2006, 2007–2008 and 2009–2010 were 69%, 71%, 71% and 72%, respectively, calculated as the number of examined adults divided by the total number selected for the sample [12,13,14,15]. There were 22,130 adults aged ≥19 years who attended a mobile examination center (MEC). We excluded pregnant women (variable RIDEXPRG = 1) (*n* = 700), participants with unreliable 24-h dietary recalls (variables DR1DRSTZ and DR2DRSTZ = 2) or no dietary recalls completed (variables DR1DRSTZ and DR2DRSTZ = 5) (*n* = 1244), those with incomplete information on the consumption of ready-to-eat cereals (RTE) (*n* = 1852) and those who had incomplete information (*n* = 12) or extreme intakes (*n* = 1) on the use of supplements containing folic acid. A total of 3809 participants were excluded as shown in Figure 1. This left 18,321 participants (82.8% of examined, unweighted) for analyses of nutrient intakes.

To compare excluded and included populations, we used 8-year combined examination weights (variable WTMEC2YR/4) and SAS (Release 9.2; SAS Institute, Cary, NC, USA) and SUDAAN statistical software (Release 9.0; Research Triangle Institute, Research Triangle Park, NC, USA) to account for the complex sampling design. Compared to the adults excluded, the 18,321 participants in our study population were more likely to be older (geometric mean, 46.9 years compared to 41.9 years, *p* < 0.001), non-Hispanic whites (72.1% compared to 58.2%, *p* < 0.0001) and supplement users (37.1% compared to 29.9%; *p* *<* 0.0001).

### 2.2. Intakes of Folic Acid and Vitamin B12

To estimate usual daily nutrient intakes, we used self-reported information from two 24-h dietary recalls and questionnaire responses regarding supplement use in the past 30 days from NHANES 2003–2004, 2005–2006, 2007–2008 and 2009–2010. Dietary intake data in the previous 24 h were collected in person at a MEC (Day 1) and by telephone 3–10 days (Day 2) after MEC participation using the U.S. Department of Agriculture Automated Multi-Pass Method, described in detail elsewhere [16,17]. We estimated folic acid intake from foods using the U.S. Department of Agriculture Food and Nutrient Databases for Dietary Studies for NHANES 2003–2004 (FNDDS Version (v.) 2.0), 2005–2006 (FNDDS v. 3.0), 2007–2008 (FNDDS v. 4.1) and 2009–2010 (FNDDS v. 5.0) [18,19,20,21].

Although information on supplement intake was also available through 24-h dietary recalls beginning in 2007, to be consistent with prior years in our analysis, we analyzed only supplement use information obtained during the household interview. Participants responded to questions regarding their supplement intake during the past 30 days. For each supplement, participants were asked about the number of days of consumption and the amount consumed per day. Interviewers recorded the name of each product from the label if available or, if not available, from the participant report. After the interview, trained nutritionists matched product names to known dietary supplements. Label information was recorded for each product, including the ingredients, amount of each ingredient (nutrient dose) in a serving, the unit for that amount (e.g., µg or mg) and the number of units (e.g., tablets or ounces) in a serving (serving-size quantity). Missing or unknown information was replaced by default values as described in the NHANES documentation for each survey [22,23,24,25]. Supplement consumers were defined as those who reported consumption of supplements containing folic acid in the past 30 days. For each individual, a nutrient intake per supplement was estimated from the ingredient unit for the supplement, the amount of the supplement consumed on each consumption day divided by the amount in one serving (serving-size quantity), the nutrient dose in one serving and the number of days that the supplement was consumed in the past 30 days. For each individual, folic acid intake per supplement was estimated from the ingredient unit for the supplement, the amount of the supplement consumed on each consumption day and the number of days the supplement was consumed in the past 30 days, all divided by the amount in one serving (serving-size quantity). Amounts of folic acid consumed in each supplement were summed across all supplements consumed over the past 30 days and then divided by 30 to yield an average total daily intake of folic acid from supplements. Using the ingredient quantity variable, DSDQTY, we coded and assessed for consumption of a supplement that contained 800 µg or more of folic acid per dose. We also assessed for consumption of more than one supplement containing folic acid.

Total daily folic acid intake was calculated by adding the daily intake of folic acid from fortified food on the respective day to the average daily intake of folic acid from supplements. Intakes of folic acid from fortified food and supplements were expressed in micrograms of folic acid.

### 2.3. Covariates

**Figure 2:**
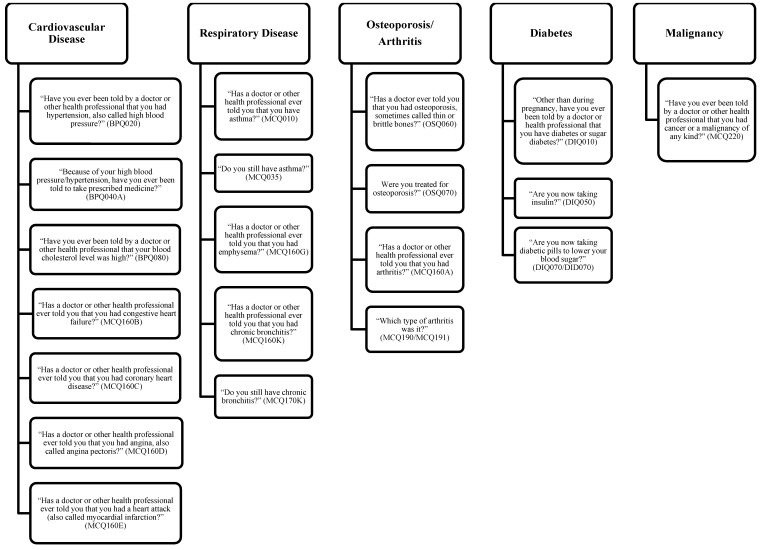
Self-response interview questions used to identify participants with chronic medical conditions: National Health and Nutrition Examination Survey (NHANES), 2003–2010.

Questionnaire information included sex, age, race/ethnicity and chronic medical conditions. We categorized adults into three age groups defined as those aged 19–39 years, 40–59 years and ≥60 years. Non-Hispanic blacks and all Hispanics (including Mexican Americans) were oversampled in NHANES, and we categorized adults into three race/ethnicity category: non-Hispanic white, non-Hispanic black and Mexican American. Other race/ethnicity groups were not reported separately due to the small sample size, but were included in the denominators for overall analysis and analysis of other sociodemographic characteristics. We also categorized participants by the presence of having none or ≥1 chronic medical conditions. Figure 2 shows the survey questions used to identify individuals with chronic medical conditions. We tried to be exhaustive in using multiple questions to identify anyone with at least one chronic medical condition. Anyone who answered yes to any of the questions in Figure 2 would be defined as having at least one chronic medical condition.

### 2.4. Folic Acid Sources and Consumption Groups

Among non-pregnant U.S. adults with usual daily folic acid intake exceeding the UL, we assessed their folic acid sources of consumption and also classified participants into four mutually-exclusive folic acid consumption groups based on ECGP consumption, as previously described elsewhere [5]. The ECGP alone group included adults who only consumed folic acid fortified foods (excluding those who consumed folic acid from RTEs or took supplements containing folic acid (SUP)). The ECGP + RTE group included adults who consumed only folic acid fortified foods and RTEs (excluding those who took SUP). The ECGP + SUP group included adults who consumed both folic acid fortified foods and supplements (excluding those who consumed RTEs). The ECGP + RTE + SUP group included adults who consumed all three sources of folic acid: fortified foods, RTEs and supplements.

### 2.5. Folic Acid Supplement Intake Groups

Among non-pregnant U.S. adults with usual daily folic acid intake exceeding the UL who took supplements, we assessed different intakes of SUP based on average daily folic acid intakes from supplements in two ways. One grouping categorized those with intakes ≤400 µg and those with intakes >400 µg, since most multivitamins contain 400 µg of folic acid. Another grouping categorized those with intakes ≤800 µg and those with intakes >800 µg, since most prenatal vitamins contain 800 µg of folic acid. We also categorized adults by the number of SUP taken (one *vs*. >1) and assessed proportions of adults who took a SUP that contains ≥800 µg/dose.

### 2.6. Statistical Analyses

We analyzed data from two 24-h dietary recalls with the Software for Intake Distribution Estimates (PC-SIDE), Version 1.02 (2003; Department of Statistics, Iowa State University, Ames, IA, USA), which fits a measurement error model to estimate usual nutrient intakes. Data from a single 24-h dietary recall or from the mean of two 24-h recalls do not account for within-individual day-to-day variation in diet and between-individual variation [26,27,28]. PC-SIDE selects for the best transformation to normalize the data, adjusting for nuisance variables (e.g., age, race/ethnicity or the day of the week the 24-h dietary recall was reported) and accounting for within and between individual variations in nutrient intake.

We used PC-SIDE to estimate the proportion of participants with usual daily intake of folic acid exceeding the UL adjusting for sex, age, race/ethnicity, interview method (in person or by phone) and the day of the week on which the 24-h dietary recall was reported, either in person or by phone. We used PC-SIDE to estimate the best linear unbiased predictor of usual daily folic acid intake for each individual. We used these estimates to classify individuals into two groups based on whether their usual daily folic acid intake exceeded the UL or not. Standard errors (SEs) were estimated by using a set of 123 jackknife repeated replication weights with PC-SIDE. Jackknife replication weights were calculated by using a combination of dietary weights based on the first 24-h dietary recall.

We used SAS and SUDAAN statistical software to estimate proportions and 95% confidence intervals (CI) of both adults with usual daily folic acid intake exceeding the UL and those with intake not exceeding the UL, stratified by sociodemographic characteristics (*i.e.*, covariates: sex, age, race/ethnicity, supplement use, having ≥1 chronic medical conditions). We also stratified by age/sex combined among those with usual daily folic acid intake exceeding the UL. Among the participants with usual daily folic acid intake exceeding the UL, we estimated proportions and 95% CI by folic acid sources of consumption, folic acid consumption groups and folic acid supplement intake groups, stratified by age/sex combined. Using PC-SIDE, we calculated the distributions (e.g., medians (interquartile ranges (IQRs))) of usual daily folic acid intake among adults with intake exceeding the UL by sex, age and race/ethnicity. We used 8-year combined 1-day dietary sampling weights (variable WTDR1D/4) for all analyses. Degrees of freedom (DF) and relative standard errors (RSE) were used to assess the representativeness and statistical reliability as recommended by NCHS [29]. We tested for differences in proportion distributions using a chi-square test and tested differences in the median usual daily intake of folic acid within subgroups (*i.e.*, sex, age, race/ethnicity groups) using a *z*-test. When DF and RSE permitted, we reported proportional differences between subgroups of sex, age, race/ethnicity, folic acid consumption groups and folic acid supplement intake groups using pairwise *t*-tests. We considered a *p*-value < 0.05 as statistically significant.

## 3. Results

### 3.1. General Characteristics of Study Participants

Baseline characteristics are shown in Table 1 for our 18,321 participants. Of non-pregnant U.S. adults, 2.7% (95% CI: 1.5–3.9) had usual daily folic acid intake exceeding the UL. Those with usual daily folic acid intake exceeding the UL were more likely to be female (62.2% compared to 51.9%; *p* = 0.001), non-Hispanic white (86.3% compared to 72.2%; *p* < 0.0001), supplement users (98.5% compared to 35.7%; *p* < 0.0001) or have at least one chronic medical condition (73.9% compared to 60.3%; *p* < 0.0001) compared to those with intake not exceeding the folic acid UL. The distributions of age groups were significantly different among those with usual daily folic acid intake exceeding the UL compared to those with intake not exceeding the UL. Those aged ≥60 years made up a significantly greater proportion among those with usual daily folic acid intake exceeding the UL compared to those with intake not exceeding the UL (34.4% *vs*. 24.8%, *p* < 0.02). Among those with usual daily folic acid intake exceeding the UL, 20.8% (95% CI: 15.5–26.2) were women aged 19–39 years and 19.9% (95% CI: 14.3–25.5) were women aged ≥60 years. Women aged ≥60 years made up a statistically higher percentage among those with usual daily folic acid intake exceeding the UL compared to those with intake not exceeding the UL (19.9% compared to 13.8%; *p* < 0.05).

### 3.2. Adults with Usual Daily Intake of Folic Acid Exceeding the UL

Results onward apply only to those adults with usual daily folic acid intake exceeding the UL (*n* = 296). Among those with usual daily folic acid intake exceeding the UL, by folic acid sources of consumption, 99.7% consumed ECGP (95% CI: 99.2–100.0), 61.2% consumed RTEs (95% CI: 53.8–68.5) and 98.5% (95% CI: 96.7–100.0) took SUP. By the four mutually-exclusive folic acid consumption groups, no one consumed ECGP alone, 1.5% consumed ECGP + RTE, 38.8% (95% CI: 31.4–46.2) consumed ECGP + SUP and 59.7% (95% CI: 46.8–67.3) consumed ECGP + RTE + SUP.

Among those with usual daily folic acid intake exceeding the UL who took folic acid supplements, 86.6% (95% CI: 81.7–91.6) took on average >400 µg of folic acid daily from supplements, 63.4% (95% CI: 57.1–69.8) took >800 µg folic acid daily from supplements, 52.5% (95% CI: 45.5–59.5) took more than one SUP and 46.6% (95% CI: 38.4–54.7) took a SUP containing ≥800 µg/dose. Women were more likely to take on average >400 µg of folic acid daily and >800 µg of folic acid daily from supplements than were men (92.8% compared to 73.0%, *p* < 0.001; 73.4% compared to 44.4%, *p* < 0.001; respectively). Among women aged 19–39 years, 98.4% (95% CI: 95.6–100) took on average >400 µg of folic acid daily from supplements. Among women aged ≥60 years, all took supplements containing folic acid, 91.3% (95% CI: 83.7–98.8) took >400 µg of folic acid daily and 72.9% (95% CI: 59.0–86.7) took >800 µg of folic acid daily. Among those aged ≥60 years, 67.8% (95% CI: 58.6–77.1) took more than one supplement containing folic acid.

Among adults with usual daily folic acid intake exceeding the UL, the median (25th–75th percentile) usual daily folic acid intake was 1477 µg/day (1298–1709 µg/day). We found no significant differences comparing median usual daily folic acid intakes by sex, age or race/ethnicity (Table 2).

## 4. Discussion

Earlier studies have reported that only 3% of U.S. non-pregnant adults have usual daily folic acid intake exceeding the UL [5,6]. To our knowledge, our study is the first to provide a detailed description of non-pregnant U.S. adults with usual daily intake of folic acid exceeding the UL. Among adults with usual daily folic acid intake exceeding the UL, the majority were women or non-Hispanic whites; none consumed ECGP alone; almost all took supplements, and among them, a majority took on average >400 µg of folic acid daily from supplements. We found that those with usual daily intake exceeding the folic acid UL were more likely to report having a chronic medical condition compared to those with intake not exceeding the UL. People with certain medical conditions (e.g., women who have had a prior pregnancy affected by neural tube defects, individuals who are taking valproic acid for seizures and individuals who are taking methotrexate for rheumatoid arthritis) may be advised by their physicians to consume high doses of folic acid, and when high doses of folic acid are taken under the care of a physician, the UL does not apply [4].

Previous findings showed that U.S. women aged 51–70 years and men aged >50 years had the highest proportions exceeding the folic acid UL compared to other age groups, and U.S. adults ≥60 years were more likely to use supplements containing folic acid compared to younger age groups [5,7]. Past studies had also showed that individuals of older age may be more likely to have usual daily intake exceeding the folic acid UL [7,30]. We found that those aged ≥60 years made up a greater proportion of those with usual daily intake exceeding the folic acid UL compared to those with usual daily intake not exceeding the UL. Of those aged ≥60 years with usual daily intake exceeding the folic acid UL who were supplement users, a majority consumed multiple supplements containing folic acid. The sample size was insufficient to further stratify supplement use by the other age groups or covariates in order to compare.

Women made up a majority among those with usual daily intake exceeding the folic acid UL, and they were more likely to take higher doses (>400 µg and >800 µg) of folic acid from supplements than were men. Among those with usual daily intake exceeding the folic acid UL, when assessing by both age and sex, we found that the largest groups consisted of women aged 19–39 years (20.8%) and women aged ≥60 years (19.9%). Since women aged 19–39 years are of childbearing age and may be taking prenatal vitamins because they are planning a pregnancy, we attempted to assess pregnancy intention in our study. Many prenatal vitamins contain 800 µg of folic acid and women intending to become pregnant who are taking these supplements can therefore easily exceed the folic acid UL. We attempted to assess the percentage of women aged 19–39 years who took a SUP containing ≥800 µg/dose, which could suggest that these women might have been following indications for folic acid supplement use, such as pregnancy intention or breastfeeding. However, the sample size was insufficient to assess these variables among the subgroup of women aged 19–39 years with usual daily folic acid intake exceeding the UL.

Development of the folic acid UL was based on evidence of masking of vitamin B-12 deficiency anemia, and thus, concerns about the folic acid UL are only applicable to those with a vitamin B-12 deficiency and not to the general population. The folic acid UL may be misinterpreted as an “upper limit”, but should only be considered a tolerable upper intake level for individuals with a vitamin B-12 deficiency. Concerns have been raised regarding other potential adverse effects of folic acid supplementation, including cancer, epigenetic changes, unmetabolized folic acid and asthma, but to date, extensive systematic reviews and meta-analyses have not consistently replicated adverse effects associated with folic acid [31,32,33,34,35,36]. The highest standard of meta-analyses comes from analyzing the individual level data, which Vollset and colleagues were able to conduct [32]. In their study, Vollset and colleagues obtained individual participant datasets that included almost 50,000 participants in 13 randomized trials of folic acid supplementation and assessed any effects on site-specific cancer rates [32]. They found that folic acid supplementation did not increase or decrease incidence of site-specific cancer rates substantially [32]. In addition to preventing neural tube defects, folic acid has been found to have other beneficial effects, e.g., prevention of stroke, reduction in autism risk in children whose mothers took folic acid early in pregnancy, near elimination of folate deficiency anemia and reduction in colorectal cancer in two large cohort studies [37,38,39,40,41]. Given the vast literature on folic acid, frequent updating of meta-analyses and continued risk assessment of folic acid is warranted.

The strengths of our study include the use of a large, nationally-representative sample of U.S. adults that included an oversampling of non-Hispanic blacks and all Hispanics (including Mexican Americans) and the use of two 24-h dietary recalls. The limitations of the study include the use of self-reported data from two different instruments. Dietary data were collected at a MEC and shortly thereafter. Supplement data were collected prior to the 24-h dietary recall, and intake was averaged over a 30-day period. The 24-h dietary recall underestimates caloric intake by ≈11% [42], but this does not indicate that micronutrient intake is also underestimated by the same amount. Actual nutrient quantities in foods may be higher or lower than the nutrient database estimates [43]. The within-person variability in usual daily intake of folic acid from supplements may be underestimated, as the average nutrient intakes from supplements do not reflect irregular patterns of intake. The dosage of folic acid from supplement labels may also be underestimated [44]. Nonresponse bias may result in an over- or under-estimation of the usual daily intake of folic acid; however, estimates of dietary intakes are weighted to account for nonresponse, thus reducing potential bias.

## 5. Conclusions

In conclusions, our findings indicate that U.S. adults with usual daily intake of folic acid exceeding the UL were mostly women, non-Hispanic whites or supplement users. Approximately one fifth of those with usual daily folic acid intake exceeding the UL were women of childbearing age, who may be taking folic acid supplements in preparation for pregnancy or breastfeeding. At the current folic acid fortification level, almost all of those with usual daily folic acid intake exceeding the UL took supplements, and no one consumed mandatorily-fortified enriched cereal grain products alone.

## Figures and Tables

**Table 1:** Sociodemographic characteristics, the presence of ≥1 chronic medical conditions and supplement use among non-pregnant U.S. adults aged ≥19 years with usual daily folic acid intake at or below the tolerable upper intake level and those above the tolerable upper intake level: National Health and Nutrition Examination Survey (NHANES), 2003–2010.

Characteristics	Those with Usual Daily Folic Acid Intake ≤1000 µg		Those with Usual Daily Folic Acid Intake >1000 µg	
	No. of Participants (Unweighted)	Weighted Percent, (95% Confidence Interval)	No. of Participants (Unweighted)	Weighted Percent, (95% Confidence Interval)
Total	18,025		296	
Sex ^1^				
Male	8886	48.1 (47.3–48.8)	126	37.8 (32.1–43.5)
Female	9139	51.9 (51.2–52.7)	170	62.2 (56.5–68.0)
Age ^1^				
19–39 years	6165	36.9 (35.6–38.3)	86	29.1 (22.3–36.0)
40–59 years	5626	38.3 (37.3–39.3)	77	36.5 (27.8–45.2)
≥60 years	6234	24.8 (23.5–26.0)	133	34.4 (26.5–42.3)
Race/ethnicity ^1^				
Non-Hispanic white	9038	72.2 (68.9–75.4)	207	86.3 (82.1–90.5)
Non-Hispanic black	3662	11.1 (9.4–12.8)	36	4.4 (2.6–6.2)
Mexican American	3344	7.8 (6.1–9.4)	27	3.4 (1.7–5.1)
Other	1981	9.0 (7.6–10.3)	26	6.0 (3.1–8.8)
Chronic medical condition ^1^				
Presence of ≥1 conditions	11,225	60.3 (58.7–61.8)	218	73.9 (66.6–81.2)
Supplement use ^1^				
Yes	5707	35.7 (34.3–37.2)	290	98.5 (96.7–100)

^1^ Significant differences in the distribution of sex, age, race/ethnicity, the presence of ≥1 chronic medical conditions and supplement use between those with usual daily intake not exceeding the tolerable upper level for folic acid (UL) and those exceeding the UL (*p* < 0.02).

**Table 2:** Median and 25th and 75th percentiles of usual daily folic acid intakes by sociodemographic characteristics among non-pregnant U.S. adults aged ≥19 years with usual daily folic acid intake above the tolerable upper intake level: National Health and Nutrition Examination Survey (NHANES), 2003–2010.

Characteristics of U.S. Adults with Usual Daily Folic Acid Intake above the Tolerable Upper Intake Level	Median (25th–75th Percentile) µg/Day
Total	1477 (1298–1709)
Sex	
Male	1385 (1256–1559)
Female	1509 (1295–1819)
Age	
19–39 Years	1464 (1320–1648)
40–59 Years	1479 (1251–1822)
≥60 Years	1429 (1276–1637)
Race/Ethnicity	
Non-Hispanic white	1482 (1316–1726)
Non-Hispanic black	1460 (1285–1711)
Mexican American	1433 (1363–1520)

## Abbreviations

The following abbreviations are used in this manuscript:

ECGP	enriched cereal grain products
LOAEL	lowest observed adverse effect level
UL	tolerable upper intake level
NHANES	National Health and Nutrition Examination Survey
RTE	ready-to-eat cereals
SUP	supplements containing folic acid

## References

[1] MRC Vitamin Study Research Group (1991). Prevention of neural tube defects: Results of the Medical Research Council Vitamin Study. Lancet.

[2] Czeizel A.E., Dudas I. (1992). Prevention of the first occurrence of neural-tube defects by periconceptional vitamin supplementation. N. Engl. J. Med..

[3] US Food and Drug Administration (1996). Food Standards: Amendment of standards of identity for enriched grain products to require addition of folic acid, final rule. Fed. Regist..

[4] Institute of Medicine (1998). Dietary Reference Intakes for Thiamin, Riboflavin, Niacin, Vitamin B6, Folate, Vitamin B12, Pantothenic Acid, Biotin, and Choline.

[5] Yang Q., Cogswell M.E., Hamner H.C., Carriquiry A., Bailey L.B., Pfeiffer C.M., Berry R.J. (2010). Folic acid source, usual intake, and folate and vitamin B-12 status in US adults: National Health and Nutrition Examination Survey (NHANES) 2003–2006. Am. J. Clin. Nutr..

[6] Tinker S., Cogswell M.E., Hamner H.C., Berry R.J. (2012). Usual folic acid intakes: A modeling exercise assessing changes in the amount of folic acid in foods and supplements, National Health and Nutrition Examination Survey, 2003–2008. Public Health Nutr..

[7] Bailey R.L., Dodd K.W., Gahche J.J., Dwyer J.T., McDowell M.A., Yetley E.A., Sempos C.A., Burt V.L., Radimer K.L., Picciano M.F. (2010). Total folate and folic acid intake from foods and dietary supplements in the United States: 2003–2006. Am. J. Clin. Nutr..

[8] National Center for Health Statistics 2003–2004 National Health and Nutrition Examination Survey (NHANES). http://wwwn.cdc.gov/nchs/nhanes/search/nhanes03_04.aspx.

[9] National Center for Health Statistics 2005–2006 National Health and Nutrition Examination Survey (NHANES). http://wwwn.cdc.gov/nchs/nhanes/search/nhanes05_06.aspx.

[10] National Center for Health Statistics 2007–2008 National Health and Nutrition Examination Survey (NHANES). http://wwwn.cdc.gov/nchs/nhanes/search/nhanes07_08.aspx.

[11] National Center for Health Statistics 2009–2010 National Health and Nutrition Examination Survey (NHANES). http://wwwn.cdc.gov/nchs/nhanes/search/nhanes09_10.aspx.

[12] National Center for Health Statistics National Health and Nutrition Examination Survey. Response rates and CPS totals. 2003–2004 Response rates. http://www.cdc.gov/nchs/data/nhanes/response_rates_cps/RRT0304MF.pdf.

[13] National Center for Health Statistics National Health and Nutrition Examination Survey. Response rates and CPS totals. 2005–2006 Response rates. http://www.cdc.gov/nchs/data/nhanes/response_rates_cps/RRT0506MF.pdf.

[14] National Center for Health Statistics National Health and Nutrition Examination Survey. Response rates and CPS totals. 2007–2008 Response rates. http://www.cdc.gov/nchs/data/nhanes/response_rates_cps/RRT0708MF.pdf.

[15] National Center for Health Statistics National Health and Nutrition Examination Survey. Response rates and CPS totals. 2009–2010 Response rates. http://www.cdc.gov/nchs/data/nhanes/response_rates_cps/RRT0910.pdf.

[16] National Center for Health Statistics National Health and Nutrition Examination Survey. Dietary Web Tutorials. Dietary Data Overview. Describe NHANES Dietary Collection Methods. http://www.cdc.gov/nchs/tutorials/dietary/SurveyOrientation/DietaryDataOverview/intro.htm.

[17] US Department of Agriculture, Agricultural Research Service USDA Automated Multiple-Pass Method. http://www.ars.usda.gov/services/docs.htm?docid=7710.

[18] US Department of Agriculture Food and Nutrient Database for Dietary Studies, 2.0. (2006). http://www.ars.usda.gov/SP2UserFiles/Place/80400530/pdf/fndds/fndds2_doc.pdf.

[19] US Department of Agriculture Food and Nutrient Database for Dietary Studies, 3.0. (2008). http://www.ars.usda.gov/SP2UserFiles/Place/80400530/pdf/fndds/fndds3_doc.pdf.

[20] US Department of Agriculture Food and Nutrient Database for Dietary Studies, 4.1 (2010). http://www.ars.usda.gov/SP2UserFiles/Place/80400530/pdf/fndds/fndds4_doc.pdf.

[21] US Department of Agriculture Food and Nutrient Database for Dietary Studies, 5.0. (2012). http://www.ars.usda.gov/SP2UserFiles/Place/80400530/pdf/fndds/fndds5_doc.pdf.

[22] National Center for Health Statistics 2003–2004 National Health and Nutrition Examination Survey. Dietary Files. 30-Day Dietary Supplement Use Documentation. http://www.cdc.gov/nchs/nhanes/nhanes2003-2004/DSQDOC_C.htm.

[23] National Center for Health Statistics 2005–2006 National Health and Nutrition Examination Survey. Dietary Files. 30-Day Dietary Supplement Use Documentation. http://www.cdc.gov/nchs/nhanes/nhanes2005-2006/DSQDOC_D.htm.

[24] National Center for Health Statistics 2007–2008 National Health and Nutrition Examination Survey. Changes to the 2007–2008 NHANES Dietary Supplement Files. http://www.cdc.gov/nchs/nhanes/nhanes2007-2008/dietary_changes.htm.

[25] National Center for Health Statistics 2009–2010 National Health and Nutrition Examination Survey. Dietary Files. 30-Day Dietary Supplement Use Documentation. http://www.cdc.gov/nchs/nhanes/nhanes2009-2010/DSQDOC_F.htm.

[26] Carriquiry A.L. (2003). Estimation of usual intake distributions of nutrients and foods. J. Nutr..

[27] Carriquiry A.L., Camano-Garcia G. (2006). Evaluation of dietary intake data using the tolerable upper intake levels. J. Nutr..

[28] Dodd K.W., Guenther P.M., Freedman L.S., Subar A.F., Kipnis V., Midthune D., Tooze J.A., Krebs-Smith S.M. (2006). Statistical methods for estimating usual intake of nutrients and foods: A review of the theory. J. Am. Diet. Assoc..

[29] National Center for Health Statistics National Health and Nutrition Examination Survey. Questionnaires, Datasets, and Related Documentation. Analytic Guidelines. Analytic and Reporting Guidelines: National Health and Nutrition Examination Survey. http://www.cdc.gov/nchs/data/nhanes/nhanes_03_04/nhanes_analytic_guidelines_dec_2005.pdf.

[30] Hamner H.C., Tinker S.C., Berry R.J., Mulinare J. (2013). Modeling fortification of corn masa flour with folic acid: The potential impact on exceeding the tolerable upper intake level for folic acid, NHANES 2001–2008. Food Nutr. Res..

[31] Crider K.S., Bailey L.B., Berry R.J. (2011). Folic acid food fortification-its history, effect, concerns, and future directions. Nutrients.

[32] Vollset S.E., Clarke R., Lewington S., Ebbing M., Halsey J., Lonn E., Armitage J., Manson J.E., Hankey G.J., Spence J.D. (2013). Effects of folic acid supplementation on overall and site-specific cancer incidence during the randomised trials: Meta-analyses of data on 50,000 individuals. Lancet.

[33] Crider K.S., Cordero A.M., Qi Y.P., Mulinare J., Dowling N.F., Berry R.J. (2013). Prenatal folic acid and risk of asthma in children: A systematic review and meta-analysis. Am. J. Clin. Nutr..

[34] Crider K.S., Yang T.P., Berry R.J., Bailey L.B. (2012). Folate and DNA methylation: A review of molecular mechanisms and the evidence for folate’s role. Adv. Nutr..

[35] Wien T.N., Pike E., Wisloff T., Staff A., Smeland S., Klemp M. (2012). Cancer risk with folic acid supplements: A systematic review and meta-analysis. BMJ Open.

[36] Qin X., Cui Y., Shen L., Sun N., Zhang Y., Li J., Xu X., Wang B., Xu X., Huo Y. (2013). Folic acid supplementation and cancer risk: A meta-analysis of randomized controlled trials. Int. J. Cancer.

[37] Huo Y., Li J., Qin X., Huang Y., Wang X., Gottesman R.F., Tang G., Wang B., Chen D., He M. (2015). Efficacy of folic acid therapy in primary prevention of stroke among adults with hypertension in China. JAMA.

[38] Surén P., Roth C., Bresnahan M., Haugen M., Hornig M., Hirtz D., Lie K.K., Lipkin W.I., Magnus P., Reichborn-Kjennerud T. (2013). Association between maternal use of folic acid supplements and risk of autism spectrum disorders in children. JAMA.

[39] Odewole O.A., Williamson R.S., Zakai N.A., Berry R.J., Judd S.E., Qi Y.P., Adedinsewo D.A., Oakley G.P. (2013). Near-elimination of folate-deficiency anemia by mandatory folic acid fortification in older US adults: Reasons for Geographic and Racial Differences in Stroke study 2003–2007. Am. J. Clin. Nutr..

[40] Gibson T.M., Weinstein S.J., Pfeiffer R.M., Hollenbeck A.R., Subar A.F., Schatzkin A., Mayne S.T., Stolzenberg-Solomon R. (2011). Pre- and postfortification intake of folate and risk of colorectal cancer in a large prospective cohort study in the United States. Am. J. Clin. Nutr..

[41] Stevens V.L., McCullough M.L., Sun J., Jacobs E.J., Campbell P.T., Gapstur S.M. (2011). High levels of folate from supplements and fortification are not associated with increased risk of colorectal cancer. Gastroenterology.

[42] Moshfegh A.J., Rhodes D.G., Baer D.J., Murayi T., Clemens J.C., Rumpler W.V., Paul D.R., Sebastian R.S., Kuczynski K.J., Ingwersen L.A. (2008). The US Department of Agriculture Automated Multiple-Pass Method reduces bias in the collection of energy intakes. Am. J. Clin. Nutr..

[43] Raper N., Perloff B., Ingwersen L., Steinfeldt L., Anand J. (2004). An overview of USDA’s Dietary Intake Data System. J. Food Compos. Anal..

[44] US Department of Agriculture USDA Dietary Supplement Ingredient Database; Release 1.

